# Screening and classification of rosehip (*Rosa canina* L.) genotypes based on horticultural characteristics

**DOI:** 10.1186/s12870-024-05031-6

**Published:** 2024-04-29

**Authors:** Kerem Mertoğlu, Melekber Sulusoglu Durul, Nazan Korkmaz, Mehmet Polat, Ibrahim Bulduk, Tuba Esatbeyoglu

**Affiliations:** 1 Department of Horticulture, Faculty of Agriculture, Usak University, Uşak, Türkiye; 2 Department of Horticulture, Faculty of Agriculture, Kocaeli University, Kocaeli, Türkiye; 3 Department of Plant and Animal Production, Ortaca Vocational School, Muğla Sıtkı Koçman University, Muğla, Türkiye; 4 Department of Horticulture, Faculty of Agriculture, Isparta University of Applied Science, Isparta, Türkiye; 5 Department of Chemical Engineering, Faculty of Engineering, Afyon Kocatepe University, Afyon, Türkiye; 6 Department of Molecular Food Chemistry and Food Development, Institute of Food and One Health, Gottfried Wilhelm Leibniz University, Hannover, Germany

**Keywords:** Dogrose, Phenolic compounds, Selection breeding, Natural antioxidants, Diversity

## Abstract

**Background:**

During the pandemic, the interest in colorful wild small fruits increased due to their positive effects on health. Also it has become very important to offer species with high nutritional value as fresh or processed products for human consumption due to increasing world population and decreasing arable land. In this context, we characterized the horticultural characteristics of 11 rosehip genotypes grown from seeds.

**Results:**

Citric acid was determined as the main organic acid in all the genotypes investigated. The mean values of the organic acids obtained from all the genotypes were found to be as follows: citric acid (7177 mg L^–1^), malic acid (3669 mg L^–1^), tartaric acid (1834 mg L^–1^), oxalic acid (1258 mg L^–1^), carboxylic acid (631.9 mg L^–1^), shikimic acid (157.8 mg L^–1^), ascorbic acid (155 mg L^–1^), and acetic acid (20.9 mg L^–1^). Ellagic acid was the dominant phenolic compound (90.1 mg L^–1^ – 96.2 mg L^–1^) in all genotypes. The average values obtained from all genotypes for total phenolics, total flavonoids, and antioxidant activity were 37 261 mg GAE L^–1^, 526.2 mg quercetin L^–1,^ and 93.6%, respectively. These characteristics had the lowest coefficients of variation, which indicated that all genotypes were similar regarding high biochemical with antioxidant effect. In addition, fruit width, fruit length, and fruit weight varied between 13.0 and 17.3 mm, 20.7 and 25.5 mm, and 1.4 and 2.7 g, respectively.

**Conclusions:**

The genotypes were categorized according to different purposes, such as suitability for wine production, making vinegar, etc. While the pomological characteristics were strongly positively correlated among themselves, they were generally found to be negatively correlated with the phytochemical characteristics. Categorizing genotypes according to different usage purposes can improve the agricultural and industrial application of rosehip and enhance their breeding efficacy.

## Introduction

With the increase in health awareness in society, the proportion of functional foods in the daily diet has increased due to their positive effects on health [1]. In places where contamination is low, species such as blackberry, European cranberry bush, hawthorn, strawberry tree, and rosehip that grow naturally in the wild and have various rich biochemical compounds have gained importance [2–5]. Rosehip fruits are rich in vitamins, minerals polyphenols, and organic acids, which have antioxidant effects [6–8]. These antioxidants positively influence the treatment of many cardiovascular, respiratory, and chronic diseases, infections, etc [9–11].Due to these reasons, it is integrated into the industry in different forms such as marmalade, jam, vinegar, and different parts for instance seeds or fruit flesh are used to enrich the benefits of the products or to extend their shelf life as food additives [12, 13].

Seeds of rosehip contain high levels of polyunsaturated fatty acids such as linoleic acid, linolenic acid, and arachidonic acid etc [14].Rosehip oil, consisting of alcohols, and essential oils is very valuable for the cosmetic industry. Due to its high regeneration ability and balanced composition, rosehip can be an alternative to roughage in animal grazing [15]. As rosehip has phytoremediation potential, it can prevent air pollution when used as a landscape plant [16]. So, its use on roadsides, parks and gardens will contribute to sustainability. Also, its plant with an attractive flowering form is suitable for landscape. Rosehip is an important source of income for rural people, and it also plays an important role in the recruitment of female and child labor in agricultural enterprises [17]. Thus, the cultivation of rosehip, which is in high demand for the above-mentioned reasons, should become widespread [7, 18, 19]. However, its cultivation is still quite limited around the world. In addition, conducted selection studies were mainly focused on characterizing the genotypes based on horticultural characteristics. The development of new genotypes for various production patterns is not aided in this way. So, in this study it was aimed to determine genotypes with potential for different uses, to ensure diversity in production to supply appropriate raw materials for processing in the industry.

Wild genotypes show higher morpho-biochemical diversity than cultivars [20, 21]. These genotypes have a higher frequency of genes that trigger resistance and phytochemical accumulation, due to their tolerance to natural enemies and stress factors in their environment [22]. Such genotypes need to be selected while growing species such as rosehip, which have a low genetic variation [23]. Additionally, the selection of these genotypes that are morphologically and biochemically adapted to increasingly unfavorable ecological conditions due to global climate change is crucial. In the short term, these genotypes should be used for production, and in the long term, new superior genotypes should be developed using these genotypes. To fulfill these objectives, cultivation of the genotypes that were selected and registered as cultivars has started in different parts of the world. Also, most of these genotypes are used as parents to develop new superior genotypes, and studies are continuing intensively all around the world [24–26]. Researches that combine genetic characteristics with physico-chemical characteristics are needed. Türkiye has a diverse rosehip population that grows naturally in a wide climate range from west to east. These genotypes, which adapt very well to various harsh climates and soil conditions, are a rich breeding material. Since it is highly adaptable, gives regular crop, and labor-intensive agricultural branch due to the shrub nature, it might be a complementary in agricultural enterprises, especially regarding the utilization of women and child labor.

Within the context of the current study, the physicochemical characteristics of 11 rosehip genotypes grown from seeds were determined, and the effects of these characteristics on the variation were also investigated. The genotypes were classified according to these sources of variation using multivariate analyses. Additionally, by elucidating the relationship among the characteristics, we obtained information that might be useful for breeding purposes.

## Materials and methods

### Plant materials

**Fig. 1:**
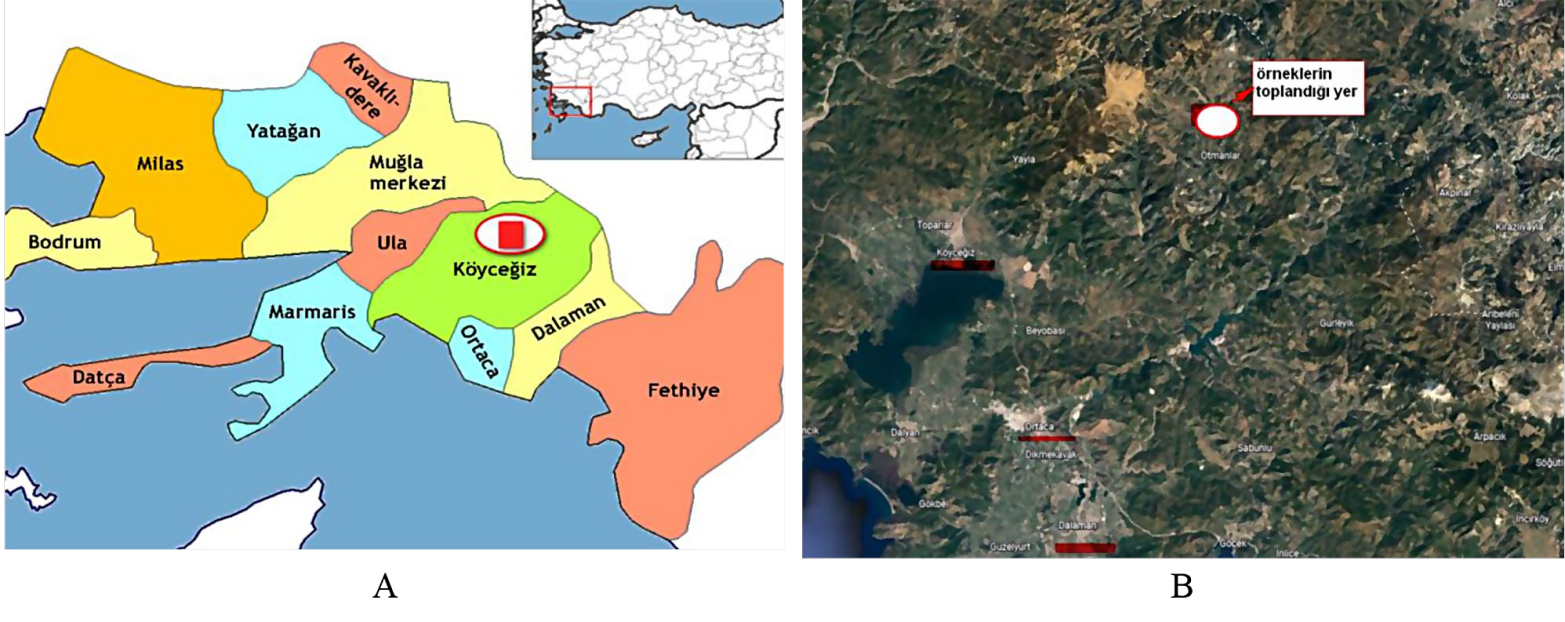
Districts and location of Mugla (A), collection site; Otmanlar Village (B). (Source: Wikipedia.org and Google Earth engine, respectively)

The study was conducted with 11 self-grown rosehip genotypes. Genotypes with dark fruit flesh color were preferred. The genotypes were collected from Otmanlar Village at 1050 altitude of Köyceğiz district, where takes place in the city of Muğla in Türkiye. Muğla is at the border of the Aegean Sea (Fig. 1) and dominated by a subtropical climate with calcareous soils.

### Harvest and determination of pomological characteristics

Fruits were picked from all sides of the shrubs to ensure homogeneous sampling. Fruits of all genotypes were hand harvested by the same person to maintain consistency of maturity degree considering the full darkness of fruit upper color [5]. Harvested fruits were immediately placed in a portable cooler (DE45) that adjusted to 4 °C with the car’s battery connection and transported to the laboratory without wasting time. Fruit width and fruit length were determined by a digital caliper (VWR-6, Milan, Italy) sensitive to 0.01 mm, and fruit weight was determined by electronic balance (Sartorius - CPA 16,001 S, Gottingen, Germany) sensitive to 0.001 g [4].

### Spectrophotometric assays

In the phytochemical analysis, all the remaining ripe fruits (at least 250 fruit) of the genotypes were collected, separated from the seeds, and converted into fruit juice using a juice extractor (Arzum, AR1060, Istanbul, Türkiye). Then, juices were filtered using a 0.45 μm membrane filter and stored at − 20 °C until the analysis. Analysis were performed when the last genotype was harvested. Before the analysis, juices were centrifuged at 14,000 rpm for 5 min. These extracted juices were used for the all phytochemical analysis.

Total phenolics (TP): TP was evaluated using the Folin-Ciocalteu assay as described and modified according to Lola-Luz et al. [27]. The fruit juice was mixed with Folin–Ciocalteu reagent and distilled water at a ratio of 1:1:18 (v/v/v) and left undisturbed for 8 min, after which, 7% sodium carbonate was added. After 2 h of incubation in the dark, the absorbance of the bluish solution was measured at 725 nm (Varian, Cary 100 Bio, Melbourne, Australia). Gallic acid was used as an external standard for the calibration curve, and the results were expressed as gallic acid equivalents (GAE) per mililiter of fruit juice (mg GAE mL^–1^).

Total flavonoids (TF): The aluminum chloride colorimetric method was used for determining TF as described by Chang et al. [28]. To summarize, 50 µL of juice was placed in a 10 mL tube and mixed with 950 µL of methanol and 4 mL of distilled water. Then, 300 µL of sodium nitrite solution (5% in water) was added. After incubation, 300 µL of aluminum chloride solution (10% in water) was added, and the mixture was allowed to stand for 6 min. Next, 2 mL of sodium hydroxide solution (1 M, in water) was added, and the final volume of the mixture was made up to 10 mL with distilled water. The mixture was left undisturbed for 15 min. Spectrophotometric analyses were conducted at 510 nm. TF was calculated from the quercetin calibration curve, and the results were expressed as mg quercetin equivalent (QE) per liter.

DPPH assay: Antioxidant activity was analyzed using the DPPH method. First, 50% inhibition concentration (IC_50_) was calculated by determining percent inhibition against the sample concentrations. Then, samples were taken up to the IC_50_ value, and the ability to remove DPPH radicals was determined following the method described by Mertoğlu et al. [29]. The antioxidant activity value was calculated taking advantage of a decrease in the absorbance value using the following formula: Antioxidant activity (%) = (A_0_ − A_1_)/A_0_ × 100, where A_1_ is the absorbance of the mixture containing the sample and A_0_ is the absorbance value of the control solution without sample. The results were expressed as a percentage (%), in which 500 mg L^–1^ ascorbic acid was used as a positive control in the analysis.

### Quantification of organic acids and phenolic compounds by HPLC-UV

The samples were shaken for 1 h and centrifuged at 14,000 rpm for 15 min. The supernatant was filtered using a 0.45 μm membrane filter. The filtered juice was analyzed by an HPLC device using an Agilent 1260 liquid chromatographic system (Agilent Technologies, CA, USA) equipped with the Chemstation software (version Rev. B.04.03), a quaternary pump, an autosampler, and a UV detector.

The organic acids were determined using an ACE-C18 column (4.6 mm × 150 mm, 5 μm; Hichrom Ltd., Theale, UK) equipped with a pre-column. The mobile phase consisted of a 10 mM aqueous solution of potassium phosphate (pH 2.2 with *ortho*-phosphoric acid) with a flow rate of 1 mL min^− 1^. The injection volume was 20 µL, and the detector was set to 245 nm for ascorbic acid and 210 nm for all other organic acids [30].

An ACE-C18 (4.6 mm × 150 mm, 5 μm; Hichrom Ltd., Theale, UK) column was used for the chromatographic separation of phenolic compounds. The mobile phase flow rate was kept constant at 1.0 mL min^− 1^. Mobile phase A was ultrapure water containing 0.1% acetic acid, whereas mobile phase B was acetonitrile containing 0.1% acetic acid. The gradient conditions were as follows: 0–3.25 min, 8–10% B; 3.25–8 min, 10–12% B; 8–15 min, 12–25% B; 15–15.8 min, 25–30% B; 15.8–25 min, 30–90% B; 25–25.4 min, 90–100% B; 25.4–30 min, 100% B. The injection volume was 10 µL, and the column temperature was maintained at 25 °C. Detection wavelengths were selected based on the wavelengths at which the phenolic compounds to be analyzed had maximum absorption. Syringic acid, protocatechuic acid, and gallic acid were detected at 280 nm; vanillic acid was detected at 225 nm, and *p*-coumaric acid was detected at 305 nm. Caffeic acid and chlorogenic acid were detected at 330 nm [31].

### Statistical analysis

We used a randomized plot experimental design to conduct this study. The pomological characteristics were measured from 25 fruits collected from each of the four sides of the plants (100 fruits for each genotype). The differences in the characteristics among the genotypes were evaluated by performing the one-way ANOVA by using Minitab (Version 17 Minitab Inc., State College, Pennsylvania, USA). Tukey (HSD) multiple comparison test was conducted to assess the differences between genotypes. The relationship between the characteristics was determined by correlation analysis and expressed with Pearson correlation coefficients. Principal component analysis (PCA) was performed to examine the interrelations among the observed set of variables to identify the similarities and differences in the characteristics. Additionally, scatter plots were generated based on the first two principal components (PC1 and PC2). Genotypes were also grouped based on characteristics investigated by using hierarchical cluster analysis (HCA). R statistical software v. 4.0.3 was used for principal component analysis, hierarchical cluster analysis and correlation analysis [32, 33].

## Results and discussion

The results of the physicochemical characteristics examined in the rosehip genotypes are presented in Table 1. Fruit width, fruit length, and fruit weight were found to vary between 13.0 and 17.3 mm, 20.7 and 25.5 mm, and 1.4 and 2.7 g, respectively, and these characteristics were significantly different among the genotypes (Table 1). The coefficient of variation calculated for fruit weight (18.9%) was higher than that calculated for fruit width (9.5%) and fruit length (7.5%). This occurred probably due to the elliptical shape of rosehip fruits. The elliptical shape of fruits belonging different rosehip genotypes was visually demonstrated, and its effects on fruit were found to be compatible with our study [34]. In studies conducted in regions with different ecological characteristics in other parts of the world, fruit width, fruit length, and the fruit weight of rosehip genotypes were found to vary within the limits of 10.4–18.4 mm, 15.1–33.8 mm, and 0.9–5.0 g, respectively [19, 23, 34]. Similar to our findings, these characteristics showed middle-to-high variation in selection studies conducted on minor fruit species [4].

**Table 1:** Descriptive statistics of pomological and chemical characteristics

Abbreviation			Unit	Minimum		Maximum	Mean ± StDev	CV (%)
General physco-chemical characteristics								
Fruit width		FrWi	mm	13.0		17.3	15.1 ± 1.4*	9.5
Fruit length		FrL	mm	20.7		25.5	23.2 ± 1.7*	7.5
Fruit weight		FrWe	g	1.4		2.7	2.0 ± 0.4*	18.9
Total phenolics		TP	mg GAE g^–1^	35.0		39.7	37.3 ± 1.4ns	3.8
Total flavonoids		TPFlvC	mg Quercetin L^–1^	457.2		625.0	526.2 ± 44.9*	8.5
Antioxidant activity		AntAc	µg Trolox m^–^	92.5		94.8	93.6 ± 0.6ns	0.7
**Organic acids**								
Citric acid		CitA	mg L^–1^	6352		8766	7177 ± 679.4*	9.5
Malic acid		MalA	mg L^–1^	2842		4583	3669 ± 637.4*	17.4
Tartaric acid		TarA	mg L^–1^	1420		2294	1835 ± 318.3*	17.4
Oxalic acid		OxaA	mg L^–1^	1010		1461	1258 ± 131.6*	10.5
Carboxylic acid		CarA	mg L^–1^	489		739.5	631.9 ± 82.0*	13.0
Shicimik acid		ShiA	mg L^–1^	135.1		182.2	157.8 ± 15.7*	10.0
Ascorbic acid		AscA	mg L^–1^	100.1		211.1	154.6 ± 30.8*	20.0
Acetic acid		AceA	mg L^–1^	10.0		30.0	20.9 ± 7.0*	33.5
**Phenolic compounds**								
Ellagic acid		EllA	mg L^–1^	90.1		96.2	92.55 ± 1.0ns	2.0
Sinapic acid		SinA	mg L^–1^	33.1		46.6	40.3 ± 4.0*	10.0
Gentisic acid		GenA	mg L^–1^	23.5		35.6	28.7 ± 3.6*	12.4
*p*-Coumaric acid		CoumA	mg L^–1^	2.3		3.4	2.8 ± 0.4*	13.2
Catechic acid		CatA	mg L^–1^	1.2		2.8	1.8 ± 0.5*	27.6
Chlorogenic acid		-	-	nd		nd	-	-
Syringic acid		-	-	nd		nd	-	-
Caffeic acid		-	-	nd		nd	-	-

StDev: Standard deviation; CV: Coefficient of variation;

*: Means statistical difference among genotypes, ns: non-significant, nd: not detected

Different parts of the rosehip plant, such as fruits, leaves, and seeds, contain high levels of biochemicals that have antioxidant effects [14]. Thus, these parts are widely used for product improvement in the food industry [13, 14]. It has been reported that by using different parts of the rosehip during process, increased the nutritional composition of products and shelf life of these products was extended due to the biochemicals with high antioxidant activity [35]. Our findings also showed that rosehip genotypes contain high levels of phenolic compounds that have antioxidant activity. TP (3.8%) and antioxidant activity (0.7%) had the lowest coefficients of variation, which indicated that all genotypes were similar regarding these characteristics; the average values obtained from all genotypes were 37 261 mg GAE L^–1^ and 93.6%, respectively. TF differed significantly among genotypes, and the values ranged from 457.2 mg QE L^–1^ to 625.0 mg QE L^–1^ (Table 1). Similar values for TP (35 430 mg GAE L^–1^ – 48 070 mg GAE L^–1^) and TFC (206 mg QE L^–1^ – 672 mg QE L^–1^) were reported by Soare et al. [6] in rosehip fruits harvested at an altitude similar to that at which our study was conducted, whereas, higher levels of TP (38 510 mg GAE L^–1^ – 79 080 mg GAE L^–1^) and TFC (287 mg QE L^–1^ – 1686 mg QE L^–1^) were reported for fruits harvested at higher altitudes [34]. As known, higher levels of phenyl propanoid enzymes are activated in plants that adapt to the typical climatic characteristics of high altitudes and trigger phytochemical accumulation [36]. As a defense mechanism against the harmful effects of high UV rays, plants produce phenolic compounds with high UV absorbing ability in epidermal tissues, in the phenylpropanoid pathway [37]. Josuttis et al. [38] reported that the quantity of phenolic acids in strawberries grown in open field were determined higher than those cultivated inside UV-blocking plastic tunnels because of exposed to higher UV that is increased at high altitudes [29]. At higher altitudes, the light intensity and the temperature difference between day and night increase. All these factors provide a higher level of synthesis of the metabolites for instance organic and phenolic acids during increased photosynthesis [39]. Thus, the accumulation of bio-chemicals with antioxidant effects per unit area is increasing.

Citric acid was detected as the main organic acid in all genotypes examined, which was similar to the results reported by Koç [40] and Igual et al. [41]. The mean values of the examined organic acids obtained from all genotypes followed the order: citric acid (7177 mg L^–1^) > malic acid (3669 mg L^–1^) > tartaric acid (1835 mg L^–1^) > oxalic acid (1258 mg L^–1^) > carboxylic acid (631.9 mg L^–1^) > shikimic acid (157.8 mg L^–1^) > ascorbic acid (154.6 mg L^–1^) > acetic acid (20.91 mg L^–1^). All examined organic acids showed statistically significant differences between the genotypes. Due to its high coefficient of variation, acetic acid (33.51%) played a key role in distinguishing among the genotypes. Carboxylic acid (12.97%), malic acid (17.35%), tartaric acid (17.35%) and ascorbic acid (19.92%) showed a moderate level of variation among the genotypes, while citric acid (9.46%), shikimic acid (9.95%), and oxalic acid (10.45%) had low variation among the genotypes. Among 25 different species, rosehip was found in the group with very rich in organic acids and it was reported that organic acids generally showed moderate variation among the genotypes [42].

Ellagic acid had a low coefficient of variation (1.95%) and was the dominant phenolic compound; its content was high (90.08–96.24 mg L^–1^) in all genotypes. Similar results were reported by Fascella et al. [7], who studied three wild rosehip species. Among the phenolic compounds examined, the levels of sinapic acid (33.14–46.56 mg L^–1^) and gentisic acid (23.48–35.64 mg L^–1^) were moderate in the rosehip genotypes, while the levels of *p*coumaric acid (2.26–3.42 mg L^–1^) and catechic acid (1.22–2.81 mg L^–1^) were low. Similar to the findings of Fascella et al. [7] in our study, chlorogenic acid, syringic acid, and caffeic acid were not detected in the rosehip genotypes.

The findings of this study were comparable to those of other studies that investigated the bioactive profile of *Rosa canina* cultivars/genotypes [6, 8, 19, 23]. Although the differences are probably mainly due to the genotypes examined, factors such as the differences in analysis methods, ecological differences between the selection areas, harvest time and type, maturity period, etc., strongly affect the final phytochemical composition of the fruits [14, 43, 44].

**Fig. 2:**
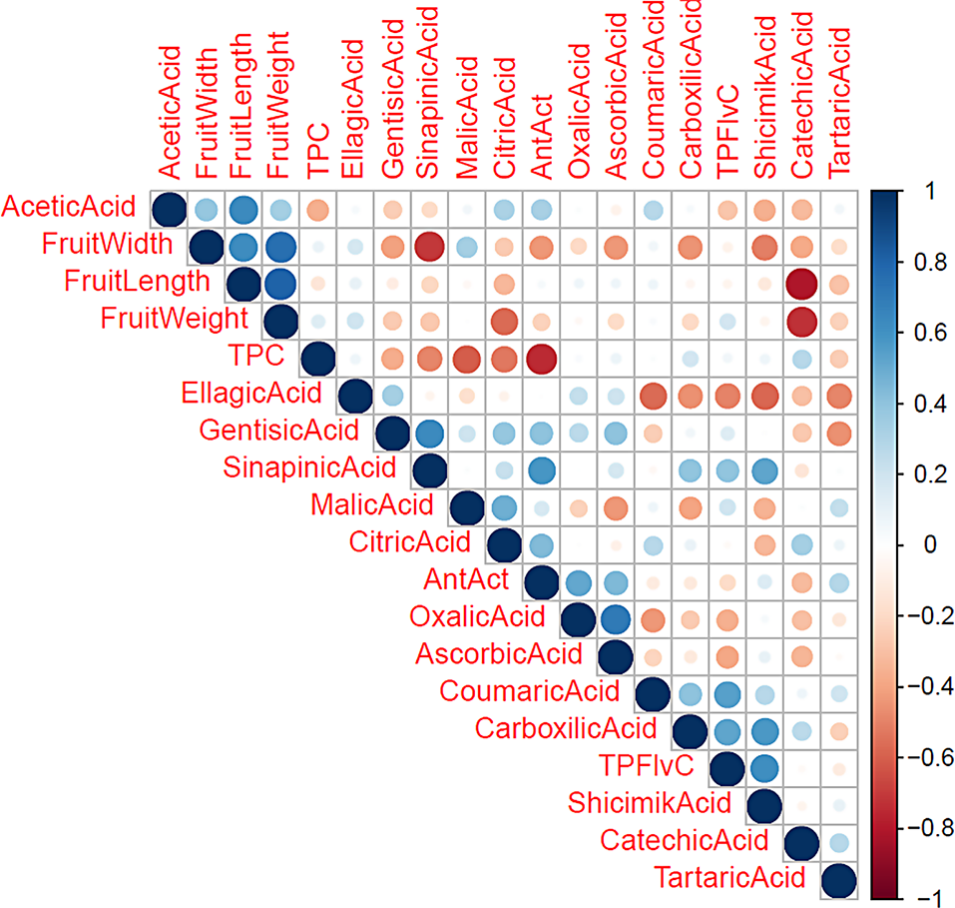
Correlations among the investigated characteristics (Darker and larger circles indicate stronger correlations. Blue represents a positive correlation, and red represents a negative correlation) (TPC: Total phenolic content, AntAct: Antioxidant activity, TPFlvC: Total flavonoid content)

The findings related to the correlations between fruit characteristics investigated are shown in Fig. 2. A high level of positive correlation was found between fruit width and fruit length (*r* = 0.63). In plants, after fertilization, the number of cells increases first, followed by the enlargement of cells. The simultaneous development of cells in the transversal and longitudinal axes during the cell expansion phase explains the strong relationship between these two characteristics. An increase in the volume leads to an increase in weight. Thus, a strong positive correlation was found between fruit weight and fruit width and also between fruit weight and fruit length respectively at (*r* = 0.75*** and *r* = 0.80***). The increase in fruit size causes a decrease in the dry matter per unit area due to an increase in the intercellular space [29]. Therefore, medium-to-high negative correlations were found between the pomological characteristics and chemical characteristics of fruits [5]. As expected, the increase in the amount of individual phenolic compounds increased the TP and TF in general. The results of this study were similar to those of studies conducted with different minor fruit species [5, 8].

Some studies reported that organic and phenolic acids with acidic characteristics are positively associated with themselves and each other [29, 43]. However, in this study, we generally found negative relationships between phenolic acids and organic acids. Additionally, an increase in organic acids caused a decrease in TP and TF. TP and TF, known for their antioxidant properties, were also found to be negatively correlated with antioxidant activity (*r* = − 0.75 and *r* = − 0.18, respectively). This relationship pattern occurred probably because the content of organic acids in rosehip fruits is substantially higher than the content of phenolic compounds. Except for carboxylic acid, all examined organic acids were positively correlated with antioxidant activity. These results indicated that the main antioxidant effect in rosehip is shaped by organic acids. Although the co-location of major quantitative trait loci (QTLs) for phenolic compounds and organic acids were reported on the same linkage groups in plum (LG1 and LG6) [45], when required, these compounds are interconvertible [46]. Therefore, these classes of compounds may sometimes show a negative relationship [44].

**Fig. 3:**
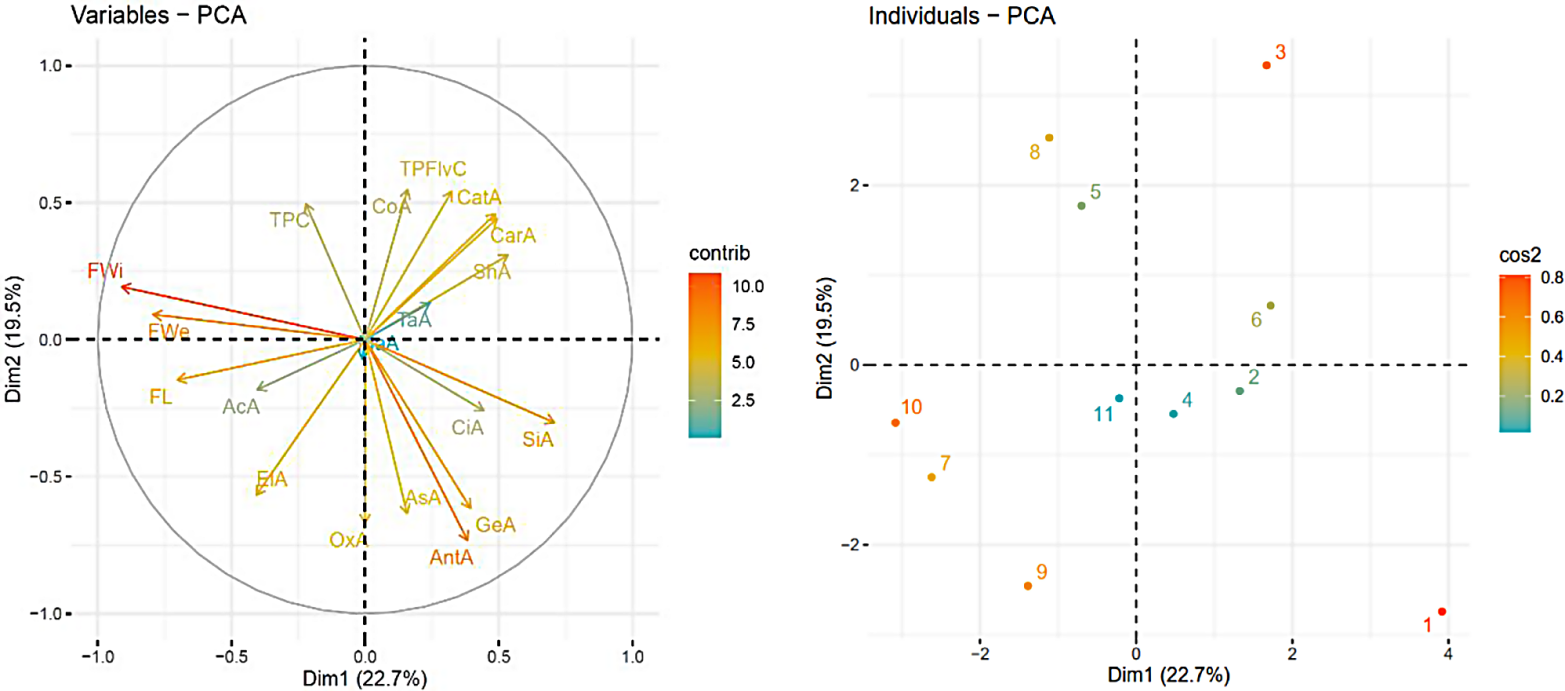
PCA-Biplot analysis of pomological and chemical characteristics (left); segregation of genotypes according to characteristics (right)

Principal component analysis (PCA) was carried out in order to assess the distribution of genotypes according to their characteristics investigated (Fig. 3). PCA was used for similar reasons in other studies conducted on different minor fruits, such as the blackberry [2], blueberry [3], European cranberrybush [43] and rosehip [6].

The results showed that the rosehip genotypes can be used for different purposes. Genotypes 5 and genotype 8 were suitable for vinegar production due to their high content of acetic acid, and genotype 1 was suitable for wine-making due to its richness in flavonoids that impart an astringent and bitter taste to the products [47]. Genotypes 3 and genotype 6 were suitable for processing in the industry because of their high organic acid content. Organic acids help maintain the stability of products by limiting the activity of microorganisms. Thus, it had been observed that microbial spoilage in products was reduced [48]. In another studies carried out both on major and minor fruit species, high organic acid content or low pH were identified as the most important criteria in order to increase antioxidant activity and long term preservation of products [29]. Genotypes 7 and genotype 10 had large fruits and might be used as parents for the development of new genotypes with large fruits in breeding studies. Large fruited genotypes with high soluble solid content/titratable acidity rate are one of the most important breeding criteria for almost all fruit species [43, 49, 50]. The biggest advantage of selection studies is that genotypes can be developed for different purposes and start crop production by cultivating them immediately.

**Fig. 4:**
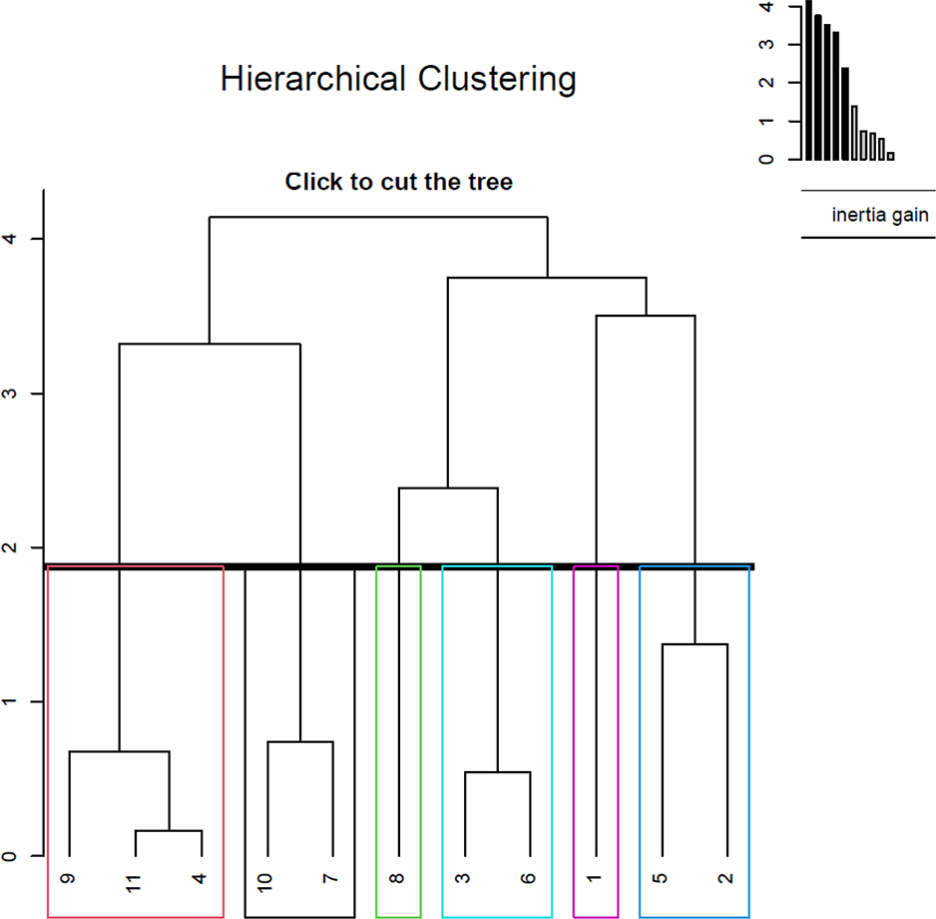
Hierarchical clustering of the genotypes studied

The results of the cluster analysis of the genotypes are shown in Fig. 4. The genotypes clustered under two main groups. Genotypes 7 and genotype 10, which formed one of the branches of Group 1, differed from the others in the pomological characteristics, whereas genotype 4, genotype 9, and genotype 11, which formed the other branch, were rich in TP. Genotype 1, constituting one of the branches of Group 2 alone, differed from others in TF. Similar results were found for genotype 8 for acetic acid and ellagic acid. Genotype 2, genotype 3, genotype 5, and genotype 6 in this group also showed a higher chemical content than the genotypes in Group 1.

## Conclusion

This was the first study to determine the physicochemical characteristics of 11 different seed-originated rosehip genotypes from Muğla province, where selection studies were not carried out before. Our results showed high variation among genotypes for various physicochemical characteristics, and thus, they might be used for different purposes. The high organic acid content of genotypes 3 and 6 might facilitate product stabilization, which can help increase the biochemical content and quality of industrial products. Genotypes 7 and 10 had a large fruit size and can be used as parents in breeding studies for developing new genotypes with large fruits and high soluble solid content/titratable acidity rate. Genotypes 5 and 8 were suitable for vinegar production due to their high content of acetic acid, and genotype 1 was suitable for wine-making due to its richness in flavonoids that give an astringent and bitter taste to products. The selection of genotypes should be continued in regions with different ecological characteristics, and the industrial suitability of genotypes investigated should be controlled by processing the fruits.

## Data Availability

All data generated or analyzed during this study are included in this published article.
